# Effects of supraspinal feedback on human gait: rhythmic auditory distortion

**DOI:** 10.1186/s12984-019-0632-7

**Published:** 2019-12-23

**Authors:** Arturo Forner-Cordero, João Pedro Pinho, Guilherme Umemura, João Carlos Lourenço, Bruno Mezêncio, Cinthia Itiki, Hermano Igo Krebs

**Affiliations:** 1 Biomechatronics Laboratory, Department of Mechatronics and Mechanical Systems of the Escola Politécnica, Universidade de São Paulo (USP), São Paulo, Brazil; 2 Instituto de Estudos Avançados of the Universidade de São Paulo (IEA-USP), São Paulo, Brazil; 3 Biomechanics Laboratory of the Escola de Educação Física e Esportes, Universidade de São Paulo (USP), São Paulo, Brazil; 4 Department of Telecommunications and Control Engineering of the Escola Politécnica, Universidade de São Paulo (USP), São Paulo, Brazil; 5 Dept. of Mechanical Engineering, MIT, Cambridge, MA02139 USA

**Keywords:** Locomotion, Subliminal gait adaptation, Gait rhythm control, Central pattern generator

## Abstract

**Background:**

Different types of sound cues have been used to adapt the human gait rhythm. We investigated whether young healthy volunteers followed subliminal metronome rhythm changes during gait.

**Methods:**

Twenty-two healthy adults walked at constant speed on a treadmill following a metronome sound cue (period 566 msec). The metronome rhythm was then either increased or decreased, without informing the subjects, at 1 msec increments or decrements to reach, respectively, a low (596 msec) or a high frequency (536 msec) plateaus. After 30 steps at one of these isochronous conditions, the rhythm returned to the original period with decrements or increments of 1 msec. Motion data were recorded with an optical measurement system to determine footfall. The relative phase between sound cue (stimulus) and foot contact (response) were compared.

**Results:**

Gait was entrained to the rhythmic auditory stimulus and subjects subconsciously adapted the step time and length to maintain treadmill speed, while following the rhythm changes. In most cases there was a lead error: the foot contact occurred before the sound cue. The mean error or the absolute mean relative phase increased during the isochronous high (536 msec) or low frequencies (596 msec).

**Conclusion:**

These results showed that the gait period is strongly “entrained” with the first metronome rhythm while subjects still followed metronome changes with larger error. This suggests two processes: one slow-adapting, supraspinal oscillator with persistence that predicts the foot contact to occur ahead of the stimulus, and a second fast process linked to sensory inputs that adapts to the mismatch between peripheral sensory input (foot contact) and supraspinal sensory input (auditory rhythm).

## Introduction

The effect of an imposed external rhythm on human motion has been extensively studied in the last decades [1, 2]. The coordination of movements following an external rhythm is named sensorimotor synchronization and it ranges from a simple finger tapping task to the skilled performance of musicians while playing a symphony. Most of the work in the analysis of rhythmic motion focused on a simple finger tapping task. This experimental paradigm has revealed important features about how humans synchronize the motion with an external pace [1]. Some of the findings on finger tapping could be extended to gait; however, due to the neuromuscular and anatomical as well as task differences, it is not possible to assume that this transference is direct [3].

Subliminal changes in rhythm caused by auditory stimulus have already been reported in finger tapping [4–7]. More recently, it has been shown that subjects modulated their responses to subliminal phase shifts of 5° [8]. These studies suggest one might expect changes in the auditory cortex interstimulus for subliminal fluctuations of 10 msec in sound interval. This is much faster than any motor evoked response and imply that the auditory system might provide a way to interrogate the motor system below conscious perception timeframes. Indeed, it appears that acoustic rhythmic stimuli are very effective to pace gait due to the strong connections between auditory and motor areas [9, 10]. In general, subcortical structures like the cerebellum and basal ganglia appear to be activated in fast rhythmic tasks (sub-second range), while cortical structures appear to be more active during longer scales [1]. This understanding is in agreement with results showing that humans are able to follow subliminal rhythm distortions in frequency and phase for arm or finger movements [6, 8, 11] with recent work showing coherence between cortico-spinal activity and leg muscles during gait [12–14].

Bank and colleagues [15], imposed changes in the gait sequence using two different manipulations: 1) perturbing the step positions, or 2) perturbing the step rhythm, in other words, requiring step length or step time adjustments. They showed that healthy elderly subjects did adapt to the projection of stepping stones and the value of the relative phase ($\varnothing =360\frac{t_{cue}-{t}_{HS}}{t_{cue}}$) was positive suggesting that the heel-strike occurred ahead of the cue time set by the metronome. Note that in their case, the perturbation (phase change) was too large to be subliminal, and the subjects adapted faster to the conditions set by the “steppingstones.” More recently, these ideas have been applied to more sophisticated setups including treadmills and virtual reality or exoskeletons [16, 17]. Strategies to employ metronomes to pace gait have also been applied in a wide range of movement disorders including stroke, cerebral palsy, Parkinson’s disease, and traumatic brain injury [16, 18–22]. These strategies could even enhance gait training for healthy older adults to prevent falls [15].

We and others have reported that subjects can alter their gait patterns based on implicit changes in gait visual feedback [23–25] and mechanical perturbation [26] with the visual distortions having a longer after-effect than mechanical perturbation [27]. Here, we expand our work on visual and mechanical perturbations and report on the effects of auditory distortion on gait [28]. More specifically, we examined experimentally the patterns of gait adaptation for imperceptible variations in the metronome rhythm. To the best of our knowledge, no other study has yet focused on understanding the effects of subliminal changes in auditory cues and their aftereffects in gait rhythm.

Synchronizing footfalls to auditory cues provides a powerful tool for training gait adaptability to environmental changes, such as those required in everyday life [9, 15]. Instantaneous and carry-over effects induced by metronome auditory cues during walking are known to affect several kinematic aspects including walking speed, cadence, stride length, and gait symmetry [15]. We hypothesized that to maintain low synchronization error (time delay between footfall and auditory cue) humans will adjust primarily the step length; the participants will maintain the synchronization error around the stimulus period even without being able to explicitly detect cue changes. However, once the metronome period stops increasing or decreasing, the participants will swiftly return to low synchronization error at the new rhythm; and there will be a carry-over effect after the phases of non-isochronous stimulus. These experiments will attempt to elucidate whether the control of a paced rhythmic task is dominated by errors in phase or frequency, which correlates to determining whether feedback or feedforward control dominates human walking. Phase error, that is, the control of the rhythm based on the error between the acoustic pacing signal and the actual motor task is a form of feedback control. The frequency error assumes that there are some kind of internal oscillators that are entrained with the external cue and that predict the behavior of the external cue, providing feedforward prediction and control of the task. If our hypotheses are proven correct, one can build a unifying hierarchical model in which a simple oscillatory central pattern generator is subservient to a model that includes peripheral and supraspinal sensorimotor control as critical elements influencing gait and its rhythmic behavior.

## Methods

### Participants

A total of 22 (6 females) undergraduate students (21.7 ± 2.2 y.o.) without motor, cognitive, sensory impairment nor previous experience in our protocol volunteered to participate in the experiment. The participants gave their signed consent. The study was approved by the Local Ethics Committee.

### Experimental design

This was a single day crossover design

### Settings and instruments

Three reflexive markers were attached to the right and left heel and to the back of the dominant hand. These were monitored by seven infrared cameras (Flex 13, Optitrack, Natural Point Inc., USA) sampled at 120 Hz, and their three-dimensional trajectories were reconstructed via commercial software Arena (Natural Point Inc., USA). A custom-made metronome based on an Arduino Uno (Arduino SpA, Italy) with a custom-made software program written in Visual Basic (Microsoft Visual Studio, USA) generated a pulse that triggered an infrared LED within the performance volume and a beep sound. The first beep of each experimental condition was synchronized to the kinematic data. Participants stepped on a treadmill (Movement LX-160, Brudden, Brazil) to perform the experiment.

### Experimental procedure

We employed a 1 msec (~ 0.6° relative phase) variation as a subliminal change. It has been shown that even musicians with good perceptual acuity were not able to detect phase changes in periodical signals smaller than 5° [8]. To confirm that subjects were unaware of the stimulus variation, they were asked to raise their hand whenever they perceived a change in the metronome period. This instruction was given verbally at the beginning of the experiment, and on 2 additional occasions (at 1/3 and 2/3 of the way through the experiment), requesting that the subjects to raise their hand if they noticed a change in rhythm.

**Fig. 1:**
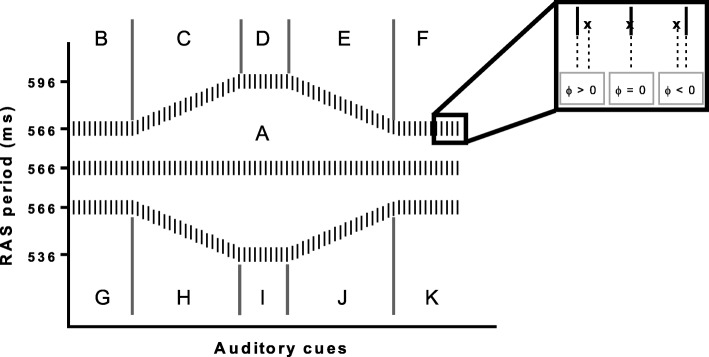
Schematic representation of the three experimental conditions subdivided into eleven phases. The insert shows the three situations that may occur when trying to synchronize footfall and beep: when the footfall (marked by an x) happens after the auditory cue (positive relative phase), when it happens with the cue (relative phase zero) and when it happens before the cue (negative relative phase)

To guarantee familiarization, participants were asked to walk on a treadmill with speed set at 1.11 m/s for five minutes. Participants were then instructed to synchronize their footfalls to the auditory cue (metronome beep) while maintaining a natural gait pattern. There were three experimental conditions, performed in a block randomized and balanced fashion, and each condition had three blocks. Between blocks, subjects were asked to continue to walk for a minute without any cueing. Figure 1 shows a schematic representation of the three conditions.

In the first experimental condition subjects were asked to walk at the sound of an isochronous metronome (566 msec) for 231 steps (condition / phase A). In the second / third condition, isochronous metronome cues were given for 59 steps (phase B / G) until an increase / decrease of 1 msec every two beeps up to 596 msec / 536 msec was introduced (phase C / H). Then, isochronous cues (596 msec / 536 msec) for 30 steps were presented (phase D / I) followed by a decrease / increase of 1 msec every two beeps back to 566 msec (phase E / J) and, finally, 26 steps of an isochronous condition (phase F / K) within that same period.

### Data analysis

The kinematic data was processed with MATLAB (2009b, MathWorks, USA) custom-made algorithms. The reflexive marker coordinates were filtered digitally by a low pass fourth order Butterworth filter with a cutoff of 12 Hz. The data was then interpolated with a spline to leave kinematic data in the same time reference. The footfall was determined by the shape of the foot markers trajectory as described elsewhere [29, 30]. Once the footfall was determined, the synchronization error was calculated as a discrete relative phase angle:

1 $$ \phi =\left(\left[\mathrm{r}\left(\mathrm{i}\right)-\mathrm{s}\left(\mathrm{i}\right)\right]/\mathrm{T}\right)\ast 360 $$

Where *ϕ* is the discrete relative phase angle, s(i) and r(i) are the stimulus (auditory cue) and response (footfall) moment; and T is the metronome period. The step length/width was obtained calculating the difference between the anterior-posterior / mediolateral forward heel coordinate and the rear one.

The means of all the steps for each trial under each phase and condition were retained for further analysis.

### Statistical procedures

The statistical procedures were conducted on SigmaStat 3.5 (Systat Software Inc., USA) and on MATLAB (Mathworks Inc., USA). After visual inspection, Shapiro-Wilk and Mauchly tests were conducted to test for normality and sphericity of the data. A one-way repeated measure analysis of variance was carried out to compare the synchronization errors and step length between the experimental phases.

The purpose of the one-way ANOVA was to make comparisons among variables of the 11 different phases as described in Fig. 1. Yet, two questions cannot be answered:

1. Whether changing rhythm induced a motor behavior change?
2. Whether similar phases occurring in a different order produce similar motor behavior? (rhythm was constant – blocks: A, B, G; rhythm increased – blocks C, J; rhythm decreased – blocks E, H; rhythm remained constant after a change – blocks D, I; and rhythm returned to the original tempo after change – blocks F, K).

We used a two-way ANOVA to try to answer these questions (condition: increase/decrease x phase). The significance level for all statistical tests was set at 5%.

To confirm that the perturbation was subliminal, we tested whether subjects raised their hand in random fashion. We performed a Chi-Squared test, assuming that subjects would do so randomly 50% of the times. The test confirmed that subjects raised their hand randomly when the metronome frequency was constant (blocks B, D, F, G, I and K).

## Results

**Fig. 2:**
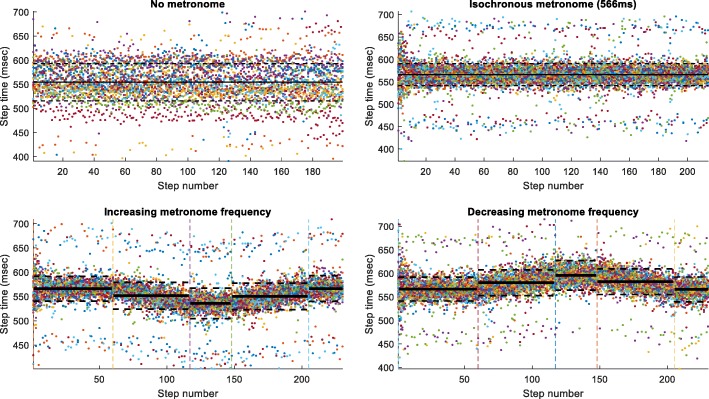
Step time (in ms) for all participants under the different experimental conditions: No Metronome, Isochronous Condition A, Increasing and Decreasing Frequency or vice-versa (see Fig. 1). The vertical lines indicate the instants of the metronome frequency transitions. The horizontal lines represent the mean (solid) and standard deviation (dashed). Top row left panel shows a wide variation representing different combinations of step length for the prescribed treadmill speed. Top row right panel shows the narrow band resulting from the introduction of the metronome beat. Lower row panels show the subliminal increase and decrease (and vice-versa) which are closely followed by the subjects

The speed of the treadmill was fixed under all conditions. The combination of average step length and speed resulted in an average walking speed equal to the treadmill speed under all conditions (see Table 1). When the subjects were asked to walk on the treadmill without any cue (no metronome), there were different combinations of step lengths and times. When the metronome was turned on, the subjects rapidly converged to the metronome rhythm as shown in Fig. 2.

**Table 1:** Mean and standard deviation of the step duration (msec) and length (m) along with the mean speed, for each metronome condition as defined in Fig. 1

CONDITION	BLOCK	Step Duration Mean ± Std (msec)	Step Length Mean ± Std (m)	Mean Speed (m/s)
1	0 (NO METRONOME)	554.38 ± 38.64	0.616 ± 0.044	1.11
	A (ISOCHRONOUS CONDITION)	565.98 ± 24.57	0.633 ± 0.029	1.12
2	B (INITIAL: CONSTANT 566 msec)	566.72 ± 24.13	0.632 ± 0.031	1.12
	C (INCREASING PERIOD TO 595 msec)	580.09 ± 23.94	0.645 ± 0.029	1.11
	D (CONSTANT: 596 msec)	595.74 ± 17.60	0.663 ± 0.023	1.11
3	E (DECREASING PERIOD TO 566 msec)	582.25 ± 21.03	0.650 ± 0.027	1.12
	F (FINAL: CONSTANT = 566 msec)	565.77 ± 23.49	0.631 ± 0.028	1.12
	G (INITIAL: CONSTANT 566 msec)	566.02 ± 25.44	0.632 ± 0.029	1.12
	H (DECREASING PERIOD TO 536 msec)	551.69 ± 27.53	0.616 ± 0.032	1.12
	I (CONSTANT: 536 msec)	535.95 ± 31.43	0.596 ± 0.036	1.11
	J (INCREASING PERIOD TO 566 msec)	550.23 ± 27.16	0.613 ± 0.033	1.11
	K (FINAL: CONSTANT = 566 msec)	566.41 ± 26.48	0.630 ± 0.031	1.11

**Fig. 3:**
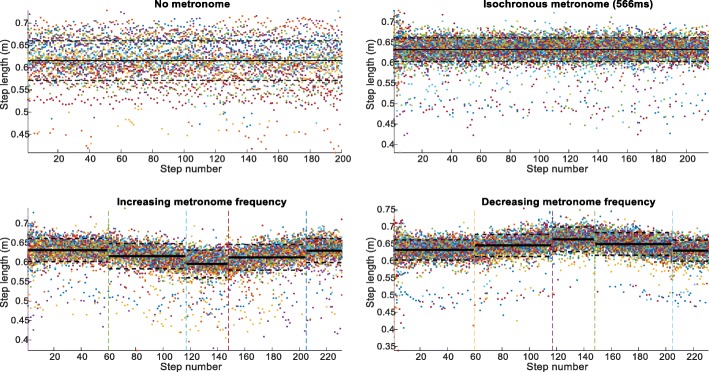
Step length (in m) for all participants under the different experimental conditions: No Metronome, Isochronous Condition A, Increasing and Decreasing Frequency or vice-versa (see Fig. 1). The vertical lines indicate the instants of the metronome frequency transitions. The horizontal lines represent the mean (solid) and standard deviation (dashed). Top row left panel shows a wide variation representing different combinations of step times for the prescribed treadmill speed. Top row right panel shows the narrow band resulting from the introduction of the metronome beat. Lower row panels show the subliminal increase and decrease (and vice-versa) which are closely followed by the subjects

When the metronome frequency increased or decreased, the subjects followed the change in the rhythm rapidly converging to the metronome rhythm. Subjects adapted the step length to the subliminal rhythm changes with the constraint of maintaining the treadmill speed, thus changing the step length accordingly (Fig. 3).

**Fig. 4:**
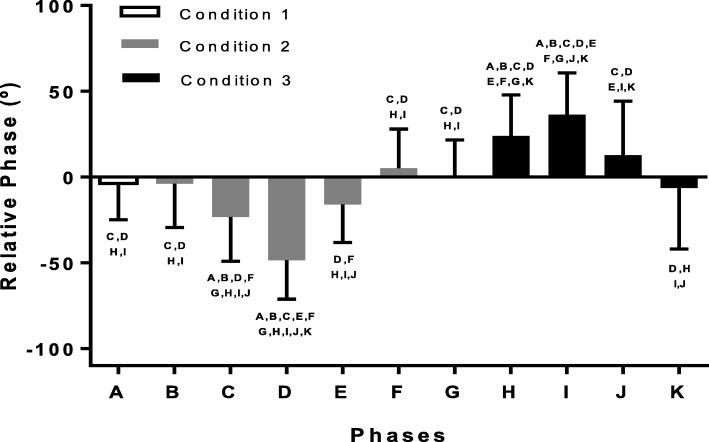
mean and standard deviation of the relative phase (expressed in degrees) in the eleven phases (A to K, as defined in Fig. 1) of the three experimental conditions (condition 1 in white, condition 2 in gray and condition 3 in black). Letters on top of the standard deviation indicate statistical differences from the referenced phase (*p* < 0.05)

The one-way repeated measures ANOVA was found to be significant (F_10,210_ = 31.260, p < 0.001). Multiple comparison procedure by Holm-Sidak method can be found in Fig. 4.

Absolute error analysis with 3 conditions and 5 groups of conditions or phases (see Table 2): 1) Isochronous initial (A, B, G); 2) inc/dec (C, H); 3) 2nd isochronous (D, I); 4) dec/inc (E, J); and 5) 3rd isochronous (F, K).

The two-way repeated measures ANOVA showed no interaction between condition and phase (F_4,84_ = 1.589, *p* = 0.185). Main effect condition was found to be non-significant (F_1,84_ = 0.059, *p* = 0.809); a significant difference was seen in the main effect phase (F_4,84_ = 18.951, *p* < 0.001). Post Hoc by Holm-Sidak method revealed a trend but no significant differences between phases (A, B, G) and (E, J) (*p* = 0.088); no differences between phases (A, B, G) and (F, K) (*p* = 0.240); phases (C, H) and (E, J) (*p* = 0.155); a trend but no significant differences for phases (C, H) and (F, K) (*p* = 0.052); and between phases (E, J) and (F, K) (*p* = 0.590). Significant differences were found between phases (A, B, G) and (C, H) (*p* = 0.002) and between phase (D, I) and phases (A, B, G) (*p* < 0.001), (C, H) (*p* < 0.001), (E, J) (*p* < 0.001) and (F, K) (*p* < 0.001).

**Table 2:** Time difference between metronome beep and heel strike [r(i) – s(i)] and relative phase in degrees, for each metronome condition as defined in Fig. 1

CONDITION	BLOCK	Time DifferenceMean ± Std (msec)	Relative Phase Mean ± Std (°)
1	A (ISOCHRONOUS CONDITION)	−7.22 ± 60.89	−4.59 ± 38.73
	B (INITIAL: CONSTANT 566 msec)	−5.08 ± 70.02	−3.23 ± 44.54
	C (INCREASING PERIOD TO 595 msec)	−36.25 ± 58.42	−22.32 ± 36.13
	D (CONSTANT: 596 msec)	− 70.88 ± 55.10	− 42.82 ± 33.28
2	E (DECREASING PERIOD TO 566 msec)	− 21.17 ± 60.82	−12.51 ± 37.72
	F (FINAL: CONSTANT = 566 msec)	10.57 ± 54.64	6.60 ± 34.72
	G (INITIAL: CONSTANT 566 msec)	−3.63 ± 58.90	−2.31 ± 37.47
	H (DECREASING PERIOD TO 536 msec)	34.42 ± 52.39	22.62 ± 34.33
	I (CONSTANT: 536 msec)	52.07 ± 51.59	34.97 ± 34.65
3	J (INCREASING PERIOD TO 566 msec)	16.30 ± 60.48	10.49 ± 39.59
	K (FINAL: CONSTANT = 566 msec)	−11.15 ± 65.23	−7.02 ± 41.70

### Subliminal rhythm change

**Fig. 5:**
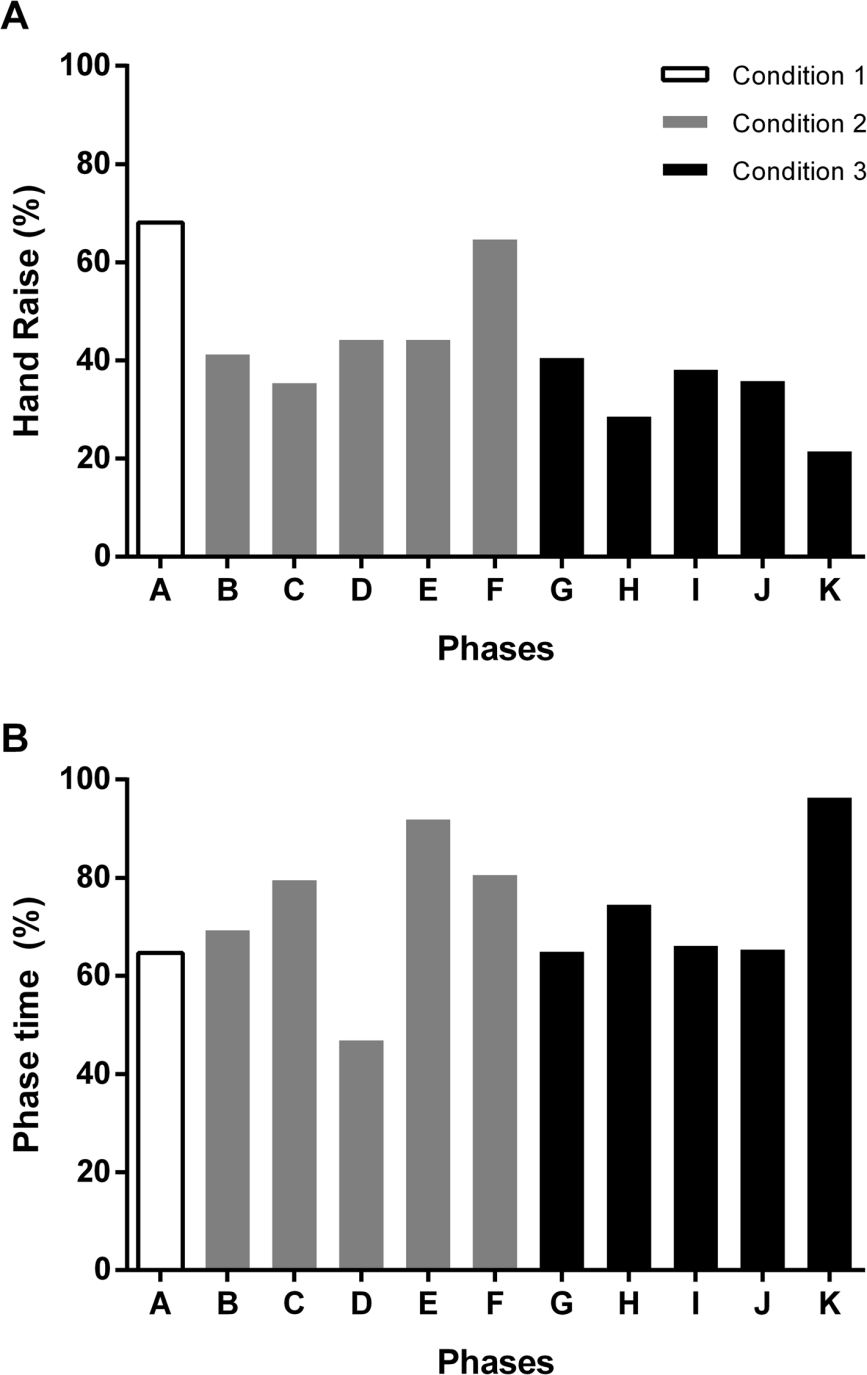
Percentage of the number of times that the hand was raised during the experiments in each block to indicate perceived changes in the metronome period (panel A) and the instant during the block when the subjects signaled the perceived changes (panel B), for the metronome phases A to K, as defined in Fig. 1

The percentage of the number of times that the hand was raised to indicate a perceived change in the metronome frequency and the relative moment when this happened is shown in panels A and B of Fig. 5. The results confirmed that the hand was raised in a random manner. The Chi-Squared test showed no difference between the number of hand raises at each block with what would be expected by chance, i.e., ${\mathcal{X}}^2$ (1, *N* = 209) = 1.303, *p* = 0.254. These results demonstrate that, indeed, it was a subliminal perturbation. Furthermore, we looked at the phase instant in which the hand was raised (see panel B in Fig. 5). It shows that when subjects identified the change in rhythm correctly in blocks C, E, H and J, they did it only in the second half of these phases when the cumulative change was larger.

## Discussion

The goal of this study was to investigate whether healthy young subjects reacted to subliminal implicit perturbations in the metronome rhythm during gait. More specifically, we examined how subliminal changes in the rhythm were integrated into the task execution.

The gait of the subjects entrained with the rhythmic auditory stimulus. When an auditory rhythm was provided to the subjects, they followed it immediately and synchronized their gait to the metronome within a few steps, as seen in Fig. 2 top row. This agrees with previous research that showed that, under verbal instruction, subjects voluntarily synchronized their step frequency to auditory cues [3, 31, 32]. Moreover, when we introduced subliminal variations in the frequency of the metronome rhythm, the subjects followed the rhythm changes, without consciously perceiving these changes, in agreement with results reported for finger or arm movement [6, 7]. Here, the rhythm changes had to comply with biomechanical constraints that are quite different from finger tapping, as the subjects had to keep up with the external constraint of a constant treadmill speed [3]. We found that the subjects subconsciously adapted the step length to maintain treadmill speed as seen in Fig. 3 bottom row. We have altered the metronome rhythm during gait on the treadmill. First, the subjects had to be entrained to a step rhythm of 566 msec. This rhythm was then maintained for the whole trial or either increased or decreased in increments of 1 msec to reach a high (536 msec) or a low (596 msec) period and, after 30 steps, the metronome returned to the initial rhythm.

When we analyzed the time error (or relative phase) between the metronome and the foot contact, we found remarkable differences among conditions. First, errors in the absolute time or relative phases in the rhythm of 566 msec were very low without any significant statistical differences, independent of the way this rhythm was presented. At this rhythm the relative phase was negative, indicating an anticipation of the foot contact to the metronome beep (phases A, B, G, K in Fig. 4). However, when the subjects returned from a low frequency condition (phase F in Fig. 4), the relative phase was positive indicating that the foot contact lagged behind the metronome beep, as if they retained some “memory” from the previous low frequency condition and maintained a longer step period.

When the metronome period increased or decreased (phases C, E, H, J), the relative phase increased because the subjects were not able to predict the next beep. In this respect, they followed the rhythm suggesting some form of feedback error correction. We observed a few features:

1. When the period increased from 566 to 596 msec (phase C), the relative phase was more negative as the foot contact occurred earlier than the beep which was being delayed at every step.
2. When the period decreased from 596 to 566 msec (phase E), the relative phase was negative, indicating that, on average, the foot contact occurred before the metronome beep despite occurring earlier at each step and suggesting a faster return to a higher frequency condition (566 msec).
3. When the period decreased from 566 to 536 msec (phase G), the relative phase became positive as the foot contact occurred after the metronome beep which occurred at increasing frequency.
4. When the period returned back to 566 msec (phase J), surprisingly, the relative phase was still positive; in spite of the increased delay in the metronome rhythm, the foot contact was occurring after the beep.
5. Subjects seemed to be unaware of the change in the metronome frequency (Fig. 5). They either indicated changes when there were none, or accurately identified changes in the second half of a block (when the cumulative change was larger). Hence, we conclude that changes in rhythm were subliminal.

These results indicate that subjects, while entrained to the first metronome rhythm, are differentially entrained to the different rhythms, perhaps suggesting preferential frequencies. We hypothesized that once the metronome period stops increasing or decreasing, the participants would swiftly return to low synchronization error at the new rhythm. However, this was not the case and the error at the different frequencies was larger when a new metronome rhythm was reached and maintained for 30 steps. The mean error/relative phase and the standard deviation was large. This occurred for both the higher (536 msec) and lower (596 msec) isochronous rhythms. We also considered that there would be a carry-over effect after the phases of non-isochronous stimulus. While the errors in the final isochronous phases (F, K) were larger, the differences were not significant even though phase F had a small mean positive relative phase error (see Fig. 4).

Our results show that the gait period is completely “entrained” with the metronome rhythm set at the beginning of the experiment. For subliminal variations in cueing, the subjects followed the rhythmic changes; however, the mean error or the mean relative phase increased as well as the standard deviation. It is very unlikely that the subjects consciously perceived changes in the period consisting of less than 5 msec. Of course, for large cumulative changes, they likely realized that they had to adapt their step length as their position on the treadmill shifted. There was clear evidence that young healthy subjects adapt to the auditory metronome beat during gait. This underscores the influence of supraspinal inputs on the purported Central Pattern Generators (CPG) of gait [33] supporting the role of a cortical loop in a hypothetical gait CPG as has been proposed by others [34–37].

This work provides behavioral support to answer two questions:

1. Does supraspinal information influence gait rhythm? The data presented here support this assumption. As the subjects walked on a treadmill at constant speed, a metronome acoustic cue was provided, and they rapidly converged to the metronome’s beat. This agrees with other experiments that measured the cortico-muscular coherence during gait and found higher coherence during certain phases of the gait cycle [12–14].
2. Subliminal changes in the metronome rhythm resulted in instantaneous adaptation of the gait rhythm of the subjects. This underscores that supraspinal sensory information influences how people walk. The acoustic cues were changed very slowly in such a way that they were not consciously perceived. Our results suggest that supraspinal inputs control or strongly influence CPGs. In this respect CPGs may sub-serve supraspinal inputs [34].

Interestingly, the errors were larger when the subjects were exposed to a rhythm different from the one they were first instructed to follow. It seems as if subjects maintained a “memory” of the first rhythm they consciously followed and only partially adapted to instantaneous subliminal variations. It suggests the possibility of a dual mechanism for entrainment: a fast process based on prediction and a slower process that tunes the gait according to an energy optimization criterion [38]. This dual mechanism may consist of two interacting processes: one slow-adapting, supraspinal oscillator with persistence that predicts the foot contact and tunes muscle activity in order to produce this contact ahead of the stimulus. In this way, it is possible to compensate for the neural delays of the foot cutaneous receptors with respect to the auditory signals that are directly connected to the brain, as this stimulation primes the motor system [39]. A second process would be directly related to the sensory inputs, and it would be rapidly adapting to the mismatch between peripheral sensory input (foot contact) and supraspinal sensory input (auditory rhythm).

Our results raised a set of interesting questions: if these changes are subliminal and cortical, will they interfere with a cognitive dual task? We have observed that this was the case with visual changes [23]. Furthermore, our results support the conjecture that an auditory “pacemaker” in combination with movement therapy in general and robotic therapies in particular might be beneficial when training rhythmic movements. This might enhance recovery after central nervous system injuries such as stroke or Parkinson’s disease [16, 17, 40, 41]. In this respect, we are presently investigating the possibility of employing this pacemaker to stimulate changes in gait rhythm and symmetry, by changing the metronome period for each footfall independently, and also to develop a comprehensive model of the integration of rhythmic sensory stimuli (visual, haptic, and auditory) for gait training.

## Conclusions

Our results showed that the gait period is strongly “entrained” with the metronome rhythm. In the isochronous conditions both at initial and low frequencies, the response (heel-strike) was slightly advanced with respect to the stimulus (metronome sound), suggesting a prediction of the sound cue. The entrainment is not completely conscious as subliminal changes in the rhythm were followed by the subjects. However, there were larger errors when the frequencies departed from the initial one. These results suggest two processes: one slow-adapting, supraspinal oscillator that predicts the foot contact to occur ahead of the sound cue, and a second fast process linked to sensory inputs that adapts to the mismatch between peripheral sensory input (foot contact) and supraspinal sensory input (sound cue).

## Data Availability

The experimental data are available.

## Abbreviations

ANOVA: Analysis of Variance
CPG: Central Pattern Generator
LED: Light Emitting Diode
Std: Standard deviation
